# Fly-scan ptychography

**DOI:** 10.1038/srep09074

**Published:** 2015-03-13

**Authors:** Xiaojing Huang, Kenneth Lauer, Jesse N. Clark, Weihe Xu, Evgeny Nazaretski, Ross Harder, Ian K. Robinson, Yong S. Chu

**Affiliations:** 1 National Synchrotron Light Source II, Brookhaven National Laboratory, Upton, NY 11973, USA; 2 Stanford PULSE Institute, SLAC National Accelerator Laboratory, Menlo Park, CA 94205, USA; 3 Center for Free-Electron Laser Science, Deutsches Elektronensynchrotron, Notkestrasse 85, 22607 Hamburg, Germany; 4 Advanced Photon Source, Argonne National Laboratory, Argonne, IL 60439, USA; 5 London Centre for Nanotechnology, University College London, London, WC1H 0AH, UK; 6 Research Complex at Harwell, Didcot, Oxfordshire OX11 0DE, UK

## Abstract

We report an experimental ptychography measurement performed in fly-scan mode. With a visible-light laser source, we demonstrate a 5-fold reduction of data acquisition time. By including multiple mutually incoherent modes into the incident illumination, high quality images were successfully reconstructed from blurry diffraction patterns. This approach significantly increases the throughput of ptychography, especially for three-dimensional applications and the visualization of dynamic systems.

As a scanning version of coherent diffraction imaging technique, ptychography provides a microscopy tool for visualizing extended specimen with the potential of delivering diffraction-limited spatial resolution by replacing image-forming optics with numerical algorithms^1^,^2^,^3^,^4^,^5^,^6^,^7^,^8^,^9^,^10^. Ptychography measures diffraction patterns when a sample scans across a confined and preferably structured incident illumination with adjacent scan positions sufficiently overlapped. The redundant information encoded in the recorded patterns is used to reconstruct the transmission function of the sample, also known as the object *O*, and the illumination function, or the probe *P*, using one-step deconvolution methods^2^,^3^,^4^,^5^, iterative projection-based^8^,^10^ or non-linear optimization algorithms^9^,^11^,^12^. Ptychography immediately found a variety of applications in two- and three-dimensional imaging operated in both transmission^7^,^8^,^13^,^14^ and Bragg geometries^15^,^16^,^17^,^18^, as well as wavefront characterization of various optics^19^,^20^,^21^,^22^,^23^,^24^,^25^,^26^,^27^.

Ptychography is typically performed in a step-scan mode, which requires the detector to wait until the specimen has been moved and settled at the target position before data acquisition starts. The overhead of step-scans (typically ~100 ms per pixel for stepper motors and ~20 ms per pixel for piezo motors) accumulates over a large number of scan positions, and is responsible for a significant overhead in data acquisition. The state-of-the-art ptychography instruments^28^,^29^ are currently dedicated for stable and efficient step-scan measurements. The effective dwell time has been pushed down to 40 *μ*s per resolution element, but each scan position still carries a 150 ms overhead time^30^, which sets the throughput limit of this scanning technique. The accumulated overhead not only slows down data acquisition, but also raises stability requirements for experimental instruments, especially for three-dimensional applications where a complete tomographical ptychography measurement take tens of hours for data collection^13^,^14^.

The scan overhead problem has been addressed in well-established scanning microscopy systems, including scanning transmission X-ray microscopy (STXM) and X-ray fluorescence microscopy (XRF), by introducing a fly-scan concept^31^,^32^, where the specimen continuously moves along the fast scan direction and the detector is synchronized and triggered over identical scan distances. A continuously moving sample however diminishes speckle visibility and creates blurry diffraction patterns. A recent algorithm development^33^ pointed out that the blurry diffraction data, caused by incoherent light sources, dynamic sample systems^34^ or background noises and point spread functions of detectors^35^, can be reconstructed by including multiple mutually incoherent probe modes representing incoherent illumination, and(or) multiple transmission functions as object modes representing different sample states, and constraining the summation of diffraction intensities from all probe and object mode pairs to intensity measurements.

Under the Born and paraxial approximations, the far-field diffraction intensity created by a monochromatic and coherent illumination on a static sample at position *j* is given by
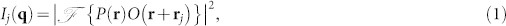
where 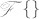 denotes Fourier transform, **q** and **r** are two-dimensional coordinates in reciprocal and real space, respectively. With a partially coherent illumination, the diffraction pattern is composed by a summation of diffraction intensities over all mutually incoherent illumination modes^36^
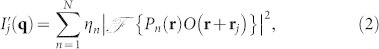
where *η_n_* is the weighting factor for the *n*th mode. In fly-scan mode, the diffraction intensity from a continuously moving sample becomes
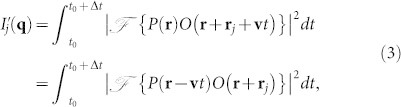
where Δ*t* is the detector dwell time and **v** is the scan speed.

Since the blurring contributions from a moving source and a moving sample are equivalent and indistinguishable on the recorded diffraction pattern, a recently reported study^37^ proposed that a static sample image can be reconstructed by attributing the measured blurry diffraction patterns from a fly-scan (Eq. 3) to partially coherent illumination (Eq. 2). This idea was previously verified with a simulated fly-scan condition by summing adjacent diffraction patterns from a regular step-scan. Here, we present an experimental demonstration of ptychography operated in fly-scan mode. We demonstrate that high-quality reconstructions are achieved with significantly reduced data acquisition time.

## Results

**Figure 1:**
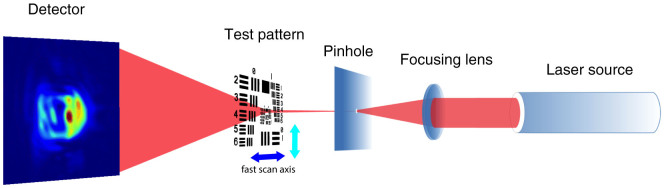
Experimental setup for fly-scan ptychography measurement. The collimated 635 nm laser is focused on a pinhole to generate the incident illumination. A test pattern was placed and scanned at a plane about 9.8 mm downstream of the pinhole. The diffraction patterns were collected by a camera placed 31 mm further downstream.

The experimental setup is shown in Fig. 1. The coherent illumination source is a fiber-coupled 635 nm laser diode. The laser beam is focused by a singlet lens onto a pinhole. The scattering wave field from this pinhole illuminates a 1951 USAF resolution target, which is driven by two Kohzu 5-phase stepper motors. Two reflection mirrors were installed on the sample stage, and the sample position was monitored by two Attocube FPS laser interferometers. An Andor Neo sCMOS detector with 6.5 *μ*m pixels was placed 31 mm downstream from the sample to collect diffraction data.

**Figure 2:**
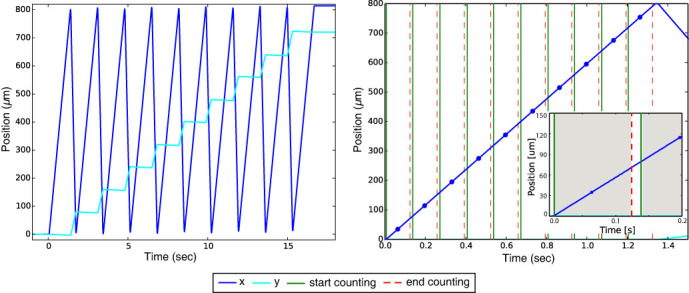
Fly-scan trajectory recorded by interferometers with 600 *μ*m/s speed and 0.12 second exposure time per frame. The motor movements along the fast (blue) and slow (cyan) scan axes are shown in the left panel. A zoomed-in view for the first scan line is displayed in the right panel. Each blue dot indicates the central location of a continuous motions during each data acquisition period. The solid green lines indicate the triggering time for the detector counting, and the dashed red lines indicate the completion time for data collection. The time interval from a solid green line to the next dashed red line defines the 0.12 second exposure time as indicated by the gray boxes in the inset, and the time interval from a dashed red line to the next solid green line is the 0.014 second dead time for detector readout.

To eliminate the “raster grid pathology” introduced by the periodical scan pattern^38^, the scan path followed a modified mesh pattern, with every second row shifted an additional 5 *μ*m in the *x* and a gradually increasing additional offset amount in the *y* direction (as shown in Supplementary Figure S1). The fast scan along the *x* axis was configured to move continuously following the defined trajectory. The scanning motor stage and the detector were synchronized by a position-compare system with high-speed synchronization capabilities (on the order of nanoseconds), toggling the trigger output to the detector when certain target positions along the trajectory have been reached. Four sets of diffraction data were collected: 0.12 second exposure time per frame for scan speeds 600 *μ*m/s, 300 *μ*m/s and 150 *μ*m/s, plus 0.135 second exposure time per frame for 450 *μ*m/s. The dead time for detector readout was set to 0.014 second. The fly-scan trajectory recorded by laser interferometers for the scan speed of 600 *μ*m/s is shown in Fig. 2.

**Figure 3:**
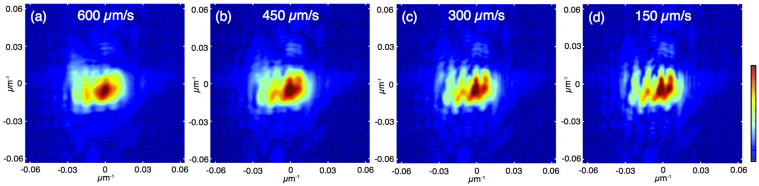
The central part of collected diffraction patterns around the same sample position with different motor speed and the same exposure time: (a) 600 *μ*m/s, (b) 450 *μ*m/s, (c) 300 *μ*m/s, (d) 150 *μ*m/s. With a faster scan speed, a larger sample area is scanned over a single exposure period, resulting in a more blurry diffraction pattern.

All scans covered the same 800 *μ*m × 800 *μ*m region, with 10 × 10, 12 × 12, 20 × 20 and 40 × 40 frames respectively. Completed datasets took 17, 24, 60 and 230 seconds, respectively. The diffraction patterns taken at an identical sample position under 4 data collection conditions are displayed in Fig. 3. The diffraction patterns exhibit a clear trend of decreasing speckle visibility with increasing scan speed, because a larger sample area traverses over the illumination within the exposure time. Consequently, a faster scan speed represents an illumination with a narrower transverse coherence length leading to more mutually incoherent modes^39^.

**Figure 4:**
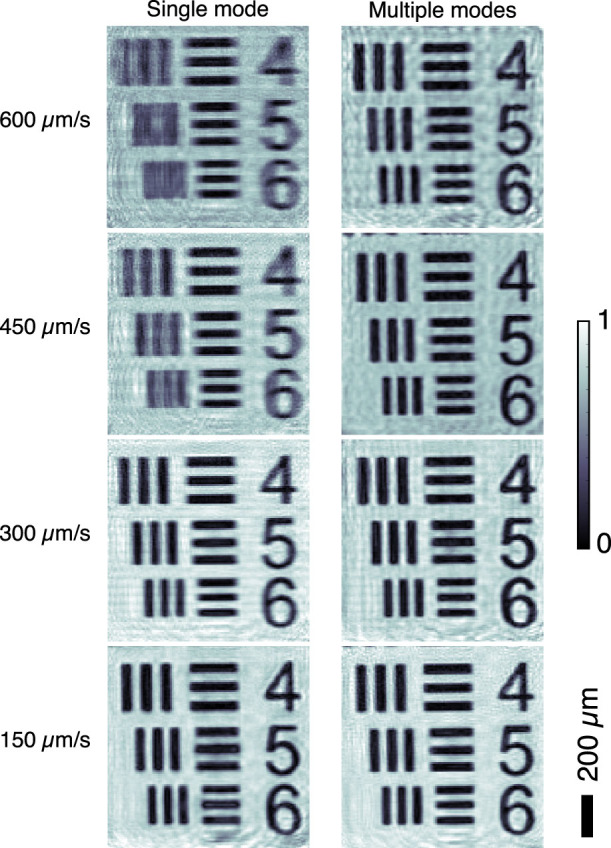
Reconstructed amplitude of the test pattern from diffraction datasets collected at different scan speeds with single (left colume) and multiple (right colume) illumination modes.

The collected diffraction patterns were fed into 500 iterations of the difference map algorithm^8^ for phase retrieval. The central position of each detector data acquisition time was used as the representative spot for each specific data frame (shown as blue dots in the right panel of Fig. 2). The object function was initiated as a random complex-valued array, and the probe was inherited from the reconstruction result of a previously acquired step-scan ptychography dataset (as shown in Supplementary Figure S2). Starting with a rough guess of the probe, such as a Gaussian function, will converge to the correct images as well with more iterations. The reconstructed amplitude images assuming fully coherent illumination, where a single probe mode is considered, are shown in the top row of Fig. 4. With 600 *μ*m/s and 450 *μ*m/s scan speeds, the reconstructions fail to produce sharp images. The images are especially smeared along the horizontal direction, which is consistent with the fast scan direction. With 300 *μ*m/s and 150 *μ*m/s scan speeds, the reconstructed images are less distorted, benefitting from less blurry diffraction patterns and higher overlapping ratios. The horizontal spatial resolutions were estimated through Gaussian fitting of line plot derivatives of the reconstructed amplitudes (shown in Supplementary Figure S4), which confirms the decrement of image quality with fast scan speeds.

The same reconstruction process was repeated with multiple probe modes. The number of probe modes *N* roughly scales inversely with transverse coherence length *σ_t_* as 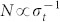, while the effective coherence length *σ_t_* is inversely proportional to the distance traversed by the sample during each exposure Δ*t* via *σ_t_* ∝ (|**v**|Δ*t*)^−1^ 34,37. As a result, a faster scan speed requires more orthogonal illumination modes to account for the effective degradation of the coherence of the measurement. However, it is not straightforward to determine the exact number of illumination modes from the above-mentioned scaling relationship. In this study, we gradually increased the number of modes until the power of the newly added mode was negligible (<1%) for the most blurry data set collected with 600 *μ*m/s scan speed. Illumination modes were initialized from the probe from single mode reconstruction by changing its amplitude by ±10% and randomly shifting it a few pixels in the horizontal direction as suggested in a previous work^34^. The recovered amplitude images reconstructed with 10 illumination modes are shown in the bottom row of Fig. 4. The blurry artifacts were successfully removed, which was verified by the estimated horizontal spatial resolutions of the reconstructed images (shown in Supplementary Figure S4), even for the data set with the fastest scan speed. For comparison, the diffraction data and reconstructed images from a 20 × 20 frames step-scan with 0.12 second exposure time are shown in Supplementary Figure S2.

**Figure 5:**
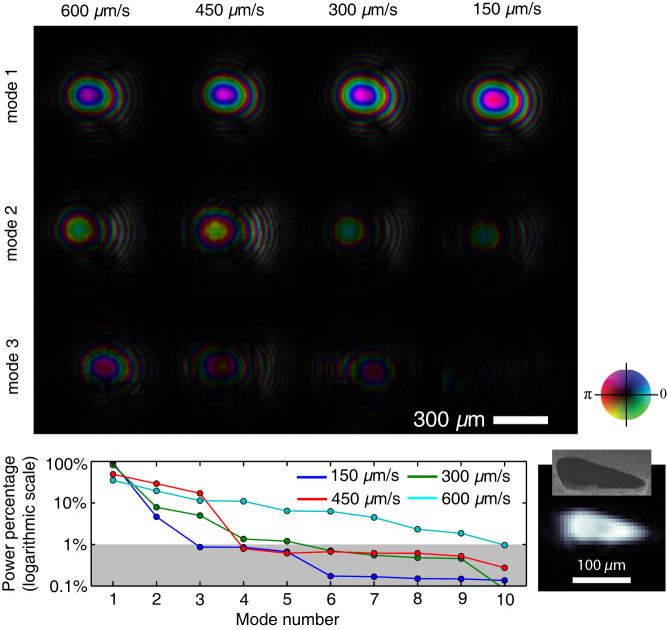
The top panel shows the three reconstructed illumination modes with the most powers for diffraction pattern collected at different scan speeds. The bottom panel shows the power percentage of the recovered modes. With increased scan speed, more illumination power is removed from primary modes and redistributed into other modes. The inset in the bottom-left corner shows the propagation of the reconstructed illumination wave back to pinhole plane. The shape and dimension are consistent with SEM measurement of the pinhole.

Fig. 5 shows the first three orthogonalized illumination modes with the most powers for all four measurement conditions. With increasing scan speed, the power of the primary mode decreases, and the intensities of other modes raise accordingly, as shown in the bottom panel of Fig. 5. At 150 *μ*m/s scan speed, over 90% of the illumination power is concentrated in the primary mode. The mode power drops below 1% after the third, the fourth, the sixth and the tenth mode for 150 *μ*m/s, 300 *μ*m/s, 450 *μ*m/s and 600 *μ*m/s scan speed, respectively. This trend is consistent with the expectation from an equivalent consideration of coherence degradation with increased scan speed. The primary illumination mode from 150 *μ*m/s scan speed dataset was used to propagate back to the pinhole pupil plane. The inset in the bottom-left panel of Fig. 5 shows the propagated amplitude at the pinhole pupil plane. The shape and dimension are consistent with the SEM measurement of the pinhole used in the experiment. The primary modes from other datasets gave virtually identical propagated images at the pinhole plane as shown in Supplementary Figure S5.

## Discussion

The scan with 20 × 20 frames and 0.12 second exposures took 292 seconds with a regular step-scan scheme, and it only took 60 seconds in fly-scan mode with 300 *μ*m/s scan speed, which represents about 5-fold enhancement of the data acquisition throughput (a similar enhancement rate was obtained with other scan speeds). Among those 60 seconds, the total exposure time took 48 seconds, the detector dead time contributed about 5.6 seconds, and the motor returning from the end of the previous scan line to the beginning of the next line accumulated about 7.4 seconds with the maximum motor speed 2.5 mm/s. With this setup, the effective photon counting time is 80% of the entire data collection time, which is a significant improvement from the state-of-the-art step-scan system (57%)^30^. This throughput rate can be further improved by reducing the detector readout time and increasing motor speed. For instance, Eiger detectors provide a continuous readout mode with 4 *μ*s dead time^40^, and a significantly higher scan speed can be adopted.

Operating ptychography in a fly-scan mode requires an identical illumination condition for each data collection period, which desires a constant scan trajectory by keeping the same scan speed along the same axis for the same time interval. This requirement rules out nonlinear scanning paths that are designed to break symmetry of the scan pattern^38^,^41^. Through simulation (summarized in Supplementary Figure S1), a modified mesh scan pattern with a varying offset from the ideal grid position at least along one direction works well to remove the periodic artifacts, while preserving the necessary properties for fly-scan.

Considering the central position of each detector acquisition period as the scan position analogous to step-scan scheme, the separation *D* of two adjacent spots are 80 *μ*m, 67 *μ*m, 40 *μ*m and 20 *μ*m for 600 *μ*m/s, 450 *μ*m/s, 300 *μ*m/s and 150 *μ*m/s respectively. The full-width-at-half-maximum (FWHM) size of the reconstructed primary mode is above 242 *μ*m (as shown in Supplementary Figure S3), which ensures sufficient overlapping rate^42^ for all measurement conditions. It is worth noting that the reconstructed horizontal spatial resolutions with single illumination mode scale linearly with the effective illumination sizes represented by the root-mean-square (RMS) widths of all illumination modes (as shown in Supplementary Figure S4). The probe dimension and the minimum overlapping condition for successful reconstruction define the upper limit of *D*, while *D* is controlled by the scan speed **v**, the exposure time Δ*t* and the detector readout time *t_r_* as *D* = |**v**|(Δ*t* + *t_r_*). As *t_r_* is determined by the detector and Δ*t* is practically optimized with the dynamic range of the detector to maximize the signal-to-noise ratio of collected data, the maximum scan speed can thus be determined.

A diode laser source was used for performing the proof-of-concept experiment. This approach can be straightforwardly reproduced and adopted to specific need by other researchers, without resorting to limited access to synchrotron sources. The presented outcome can be easily translated to X-ray experiments taking into account of the wavelength dependent parameters such as coherence properties and achievable spatial resolutions.

During the submission of this manuscript, another work was published based on a similar approach and reconstruction framework using a synchrotron X-ray source^43^. In that work, rather than using different scan speeds, the exposure time was tuned to control the overlapping ratio, where the exposure time was pushed down to Poisson statistics limit and a maximum-likelihood algorithm was used in the reconstruction process.

We experimentally demonstrate ptychography operated in a fly-scan mode. The elimination of the overhead in motor settling time was shown to improve data collection speed by a factor of five, while further enhancement is possible with hardware improvements. Diffraction patterns acquired in the fly-scan mode were successfully reconstructed to images using multiple illumination modes in the iterative phasing process to account for the equivalent loss of coherence in the measurement system. This continuous scan strategy significantly enhances the effective data collection throughput, which is highly beneficial for 3D tomography imaging and high-resolution visualization of dynamic systems.

## Methods

### Experimental setup

The coherent illumination source is a collimated 635 nm fiber pigtailed laser diode LD-635-21B (1.2 mW) from Newport, controlled with a Newport 505B laser diode driver operated at 2.1 mA. The laser beam is focused by a singlet lens with 12 mm focal length. A pinhole created on an aluminum foil is placed in the focal plane. The sample was illuminated by the scattering wave field from the pinhole. The test sample is a USAF 1951 resolution target (purchased from Precision Optical Imaging), with about 300 nm chromium deposited on a 1.5 mm thick glass substrate. The resolution target was mounted on two Kohzu 5-phase stepper motors, which offer 0.25 *μ*m half-step resolution, 0.3 *μ*m repeatability and maximum 2.5 mm/s speed. The sample position was measured by an Attocube FPS 3010 laser interferometer. This laser interferometer operates at a wavelength of 1550 nm, provides sub-nanometer resolution and is capable of tracking a moving object with a speed up to 2 m/s. The diffraction data was collected by an Andor Neo sCMOS detector with 6.5 *μ*m pixel size, placed 31 mm downstream from the sample. The detector was operated at −30°C. A total of 20 frames of dark background with the same 0.12 second exposure time were collected, averaged and subtracted from the raw data. The recorded data was 2 × 2 binned, which gave effective detector pixel size of 13 *μ*m. A 256 × 256 data array was cropped for reconstruction. With 635 nm wavelength and 31 mm detector-to-sample distance, the pixel size of reconstructed images is 5.9 × 5.9 *μ*m.

### Fly-scan control details

The Kohzu 5-phase stepper motors were controlled with two Kohzu MD-501C 5-phase micro-stepping drivers, and a Delta Tau Power PMAC motion controller with an ACC14-E digital input/output expansion board and an ACC24E-2S stepper axis expansion board. EPICS channel access is used for inter-device communication, such as detector configuration and motor positioning. Additional communication with the Power PMAC is done through a custom Python package for advanced motion script control and data collection. A Python-based script binds all of these together to complete the data acquisition system. Vital parameters, such as the scan dimensions, speed of the fast-moving axis, desired exposure time per frame, and the required read-out time of the detector, were used to generate trajectories of the motors. The controller dynamically reconfigures the hardware position-compare system. This position-compare system allows for high-speed synchronization (on the order of nanoseconds) of the detector with the sample stage, toggling the trigger output to the detector (via the ACC14-E digital I/O expansion board) when certain positions along the trajectory have been reached.

## Author Contributions

X.H., J.N.C., R.H., I.K.R. conceived the idea. X.H., K.L. and Y.S.C. designed the experiment. X.H., K.L., W.X. and E.N. performed the experiment. X.H. and K.L. analyzed the data. All authors contributed to the writing of the manuscript.

## Supplementary Material

Supplementary Information Fly-scan ptychography: supplementary materials
