# Supplementary material for: Fly-scan ptychography
Source: Sci Rep. 2015 Mar 13;5:9074. doi: 10.1038/srep09074 (PMC4357920; doi:10.1038/srep09074)
Supplement: Supplementary Information — Fly-scan ptychography: supplementary materials [file srep09074-s1.pdf]

Fly-scan ptychography: supplementary materials

Xiaojing Huang[1,*], Kenneth Lauer[1], Jesse N. Clark[2,3], Weihe Xu[1], Evgeny Nazaretski[1], Ross Harder[4], Ian K. Robinson[5,6] and Yong S. Chu[1]

[1]National Synchrotron Light Source II, Brookhaven National Laboratory, Upton, NY 11973, USA
[2]Stanford PULSE Institute, SLAC National Accelerator Laboratory, Menlo Park, CA 94205, USA
[3]Center for Free-Electron Laser Science, Deutsches Elektronensynchrotron, Notkestrasse 85, 22607 Hamburg, Germany
[4]Advanced Photon Source, Argonne National Laboratory, Argonne, IL 60439, USA
[5]London Centre for Nanotechnology, University College London, London, WC1H 0AH, UK
[6]Research Complex at Harwell, Didcot, Oxfordshire OX11 0DE, UK
*xjhuang@bnl.gov

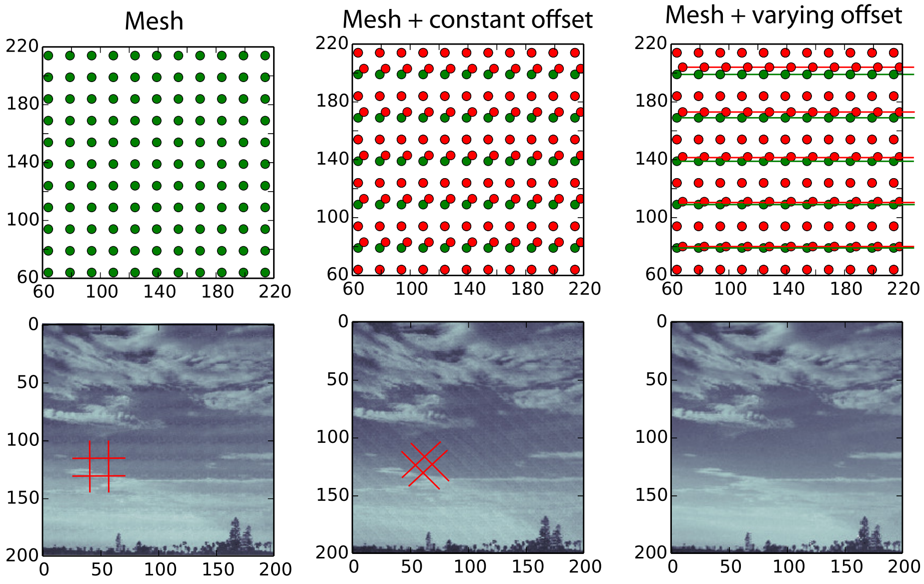

Supplementary Figure S 1: Simulation results with mesh (green dots) and modified mesh (red dots) scan patterns. The mesh pattern with a constant offset is generated by shifting the scan spots every the second line by 5 pixels in both x and y directions. The mesh pattern with varying offset is generated by shifting the scan spots every second line by 5 pixels in x direction and $n/2$ pixels in y direction, where n is the line number. Reconstructed images from the first two periodic scan patterns manifest these periodicities in the obtained images as artifacts indicated by red lines. The mesh pattern with varying offsets breaks down scan periodicity and leaves no obvious artifacts in the recovered image. Photograph was taken by X.H..

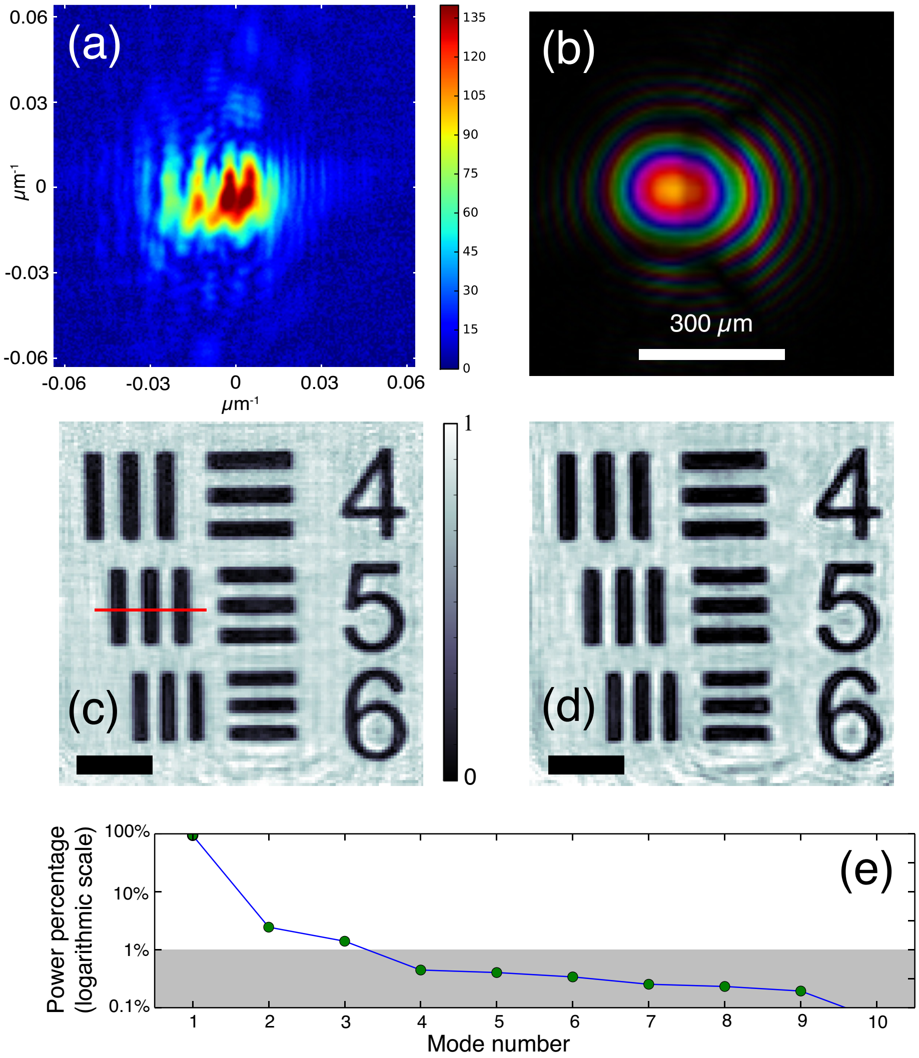

Supplementary Figure S 2: Experimental data and reconstruction results from a step scan dataset. (a) A frame of diffraction pattern at the same spot as in Fig. 3 in the main context. (b) The primary mode of reconstructed illuminations. Reconstructed amplitude with a single illumination mode (c) and multiple illumination modes (d), with 200 μm scale bar . (e) The power percentage of 10 reconstructed modes. Over 95% power is concentrated in the primary mode, which implies that the coherence property of the laser source is very good.

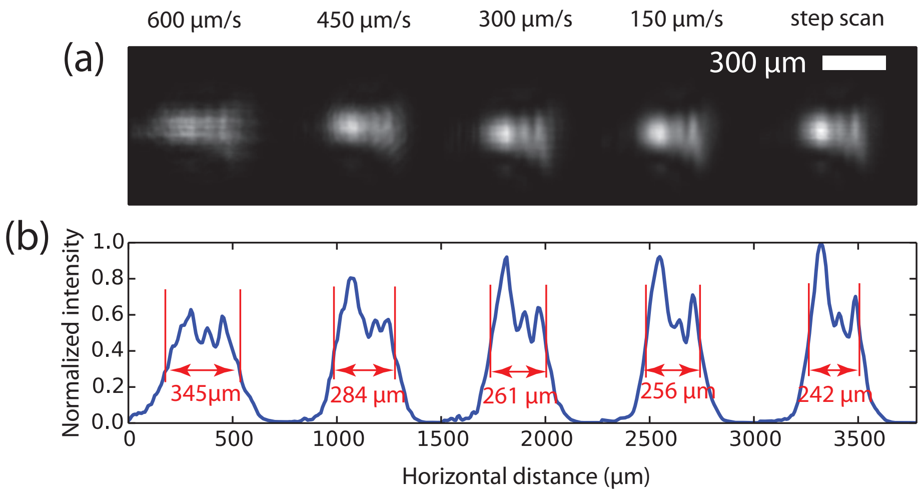

Supplementary Figure S 3: (a) The RMS intensities of all reconstructed illumination modes for all tested conditions. (b) The line plots of vertically-integrated intensities of (a). The FWHM of the RMS intensities consistently increases with faster scan speeds.

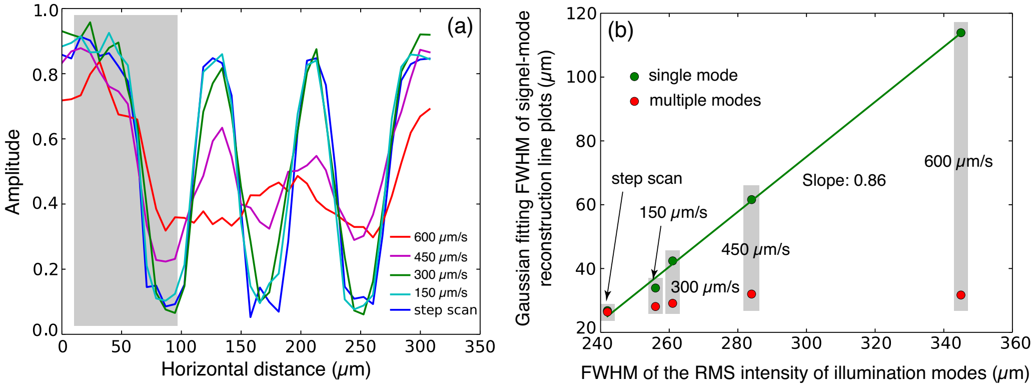

Supplementary Figure S 4: (a) The horizontal plots of reconstructed amplitudes with single illumination mode along the line indicated as the red line in Supplementary Figure S 2(c). The derivatives of the line plots at an bar edge (indicated by the gray box) were fitted with Gaussian function to estimate the reconstructed spatial resolutions. (b) shows a linear relationship between the Gaussian fitted FWHM resolution from reconstruction with single illumination mode (green dots) and the FWHM of the RMS intensities of reconstructed illumination modes. Reconstructions with multiple illumination modes (red dots) mostly recover the image resolution back.

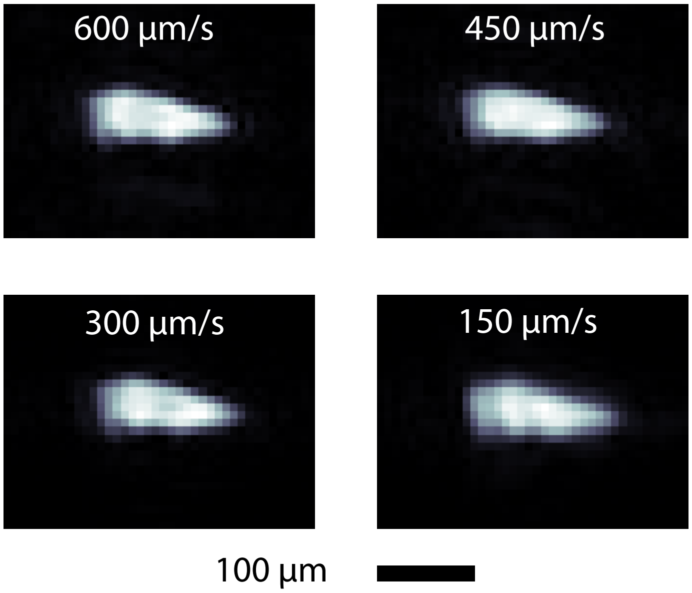

Supplementary Figure S 5: Reconstructed primary illumination modes from different scan speeds back-propragated to the pinhole plane.
